# Photodynamic Therapy for 101 Early Cancers of the Upper Aerodigestive Tract, the Esophagus, and the Bronchi: A Single-Institution Experience

**DOI:** 10.1155/DTE.5.145

**Published:** 1999

**Authors:** A. Radu, P. Grosjean, Ch. Fontolliet, G. Wagnieres, A. Woodtli, H. Van Den Bergh, Ph. Monnier

**Affiliations:** 1 Department of Otolaryngology Head and Neck Surgery CHUV Hospital Lausanne CH-1011 Switzerland; 2 lnstitute of Pathology CHUV Hospital Lausanne CH-1011 Switzerland; 3 lnstitute of Environmental Engineering EPFL Lausanne CH-1015 Switzerland

## Abstract

Cancer, when detected at an early stage, has a very good probability of being eradicated by surgery or radiotherapy. However, less aggressive treatments also tend to provide high rates of cure without the side effects of radical therapy. We report on the results of our clinical experience with photodynamic therapy (PDT) for the treatment of early carcinomas in the upper aerodigestive tract, the esophagus, and the tracheobronchial tree. Sixty-four patients with 101 squamous cell carcinomas were treated with three different photosensitizers: hematoporphyrin derivative (HPD), Photofrin II, and tetra (m-hydroxyphenyl)chlorin (mTHPC). Seventy-seven (76%) tumors showed a complete rsponse with no recurrence after a mean follow-up period of 27 months. There was no significant difference in terms of cure rates among the three dyes. However, mTHPC has a stronger phototoxicity and induces a shorter skin photosensitization than either of the other photosensitizers. There were eight major complications: three esophagotracheal fistulae after illumination with red light in the esophagus, two esophageal stenoses following 360° circumferential irradiation, and three bronchial stenoses. Illumination with the less penetrating green light and the use of a 180° or 240° windowed cylindrical light distributor render the risk of complications in the esophagus essentially impossible, without reducing the efficacy of the treatment. Therefore, PDT may be considered as a safe and effective treatment for early carcinomas of the upper aerodigestive tract, the esophagus, and the tracheobronchial tree.


*Diagnostic and Therapeutic Endoscopy*, Vol. 5, pp. 145–154
Reprints available directly from the publisher
Photocopying permitted by license only

© 1999 OPA (Overseas Publishers Association) N.V.
Published by license under
the Harwood Academic Publishers imprint,
part of The Gordon and Breach Publishing Group.
Printed in Malaysia.

### Photodynamic Therapy for 101 Early Cancers of the Upper Aerodigestive Tract, the Esophagus, and the Bronchi: A Single-Institution Experience

A. RADU[a,*], P. GROSJEAN[a], CH. FONTOLLIET[b], G. WAGNIERES[c], A. WOODTLI[c], H. VAN DEN BERGH[c] and PH. MONNIER[a]

[a]*Department of Otolaryngology, Head and Neck Surgery,* [b]*Institute of Pathology, CHUV Hospital, CH-1011 Lausanne, Switzerland;* [c]*Institute of Environmental Engineering, EPFL, CH-1015 Lausanne, Switzerland*

**Cancer, when detected at an early stage, has a very good probability of being eradicated by surgery or radiotherapy. However, less aggressive treatments also tend to provide high rates of cure without the side effects of radical therapy. We report on the results of our clinical experience with photodynamic therapy (PDT) for the treatment of early carcinomas in the upper aerodigestive tract, the esophagus, and the tracheobronchial tree. Sixty-four patients with 101 squamous cell carcinomas were treated with three different photosensitizers: hematoporphyrin derivative (HPD), Photofrin II, and tetra(m-hydroxyphenyl)chlorin (mTHPC). Seventy-seven (76%) tumors showed a complete response with no recurrence after a mean follow-up period of 27 months. There was no significant difference in terms of cure rates among the three dyes. However, mTHPC has a stronger phototoxicity and induces a shorter skin photosensitization than either of the other photosensitizers. There were eight major complications: three esophagotracheal fistulae after illumination with red light in the esophagus, two esophageal stenoses following 360° circumferential irradiation, and three bronchial stenoses. Illumination with the less penetrating green light and the use of a 180° or 240° windowed cylindrical light distributor render the risk of complications in the esophagus essentially impossible, without reducing the efficacy of the treatment. Therefore, PDT may be considered as a safe and effective treatment for early carcinomas of the upper aerodigestive tract, the esophagus, and the tracheobronchial tree.**

*Keywords:* Bronchi, Esophagus, Hematoporphyrin derivative, Tetra(m-hydroxyphenyl)chlorin, Photodynamic theraphy, Photofrin II

### INTRODUCTION

Patients with head and neck cancer are at high risk of developing additional synchronous and metachronous primary tumors, not only in the upper aerodigestive tract, but also in the esophagus and the tracheobronchial tree [1–5]. This phenomenon is attributed to the "field cancerization" concept, which suggests that the long-term carcinogen exposure leads to multiple genetic mutations throughout

* Corresponding author. Tel.: 41/21/314 27 00. Fax: 41/21/314 27 06. E-mail: Alexandre.Radu@chuv.hospvd.ch.

the aerodigestive tract epithelium [6,7]. Although still a debatable issue, thorough pretherapy screening and systematic follow-up of these patients allow the detection of a great number of second primary tumors at an early stage, i.e. *in situ* or micro-invasive [4]. Treating these early tumors with a nonmutilating and minimally invasive therapeutic modality should be highly beneficial in patients with head and neck cancer who are often in poor general condition due to a previous radical procedure directed towards the primary tumour or to concomitant diseases of the heart, lung or liver.

Photodynamic therapy (PDT), which is based on the combined effects of a photosensitizing drug and visible light, has the potential to cure such early cancers [8–15]. This relatively new therapeutic modality has the distinct advantage that it can be repeated multiple times without inducing any resistance or cumulative toxicity [16]. Photodynamic therapy can also be utilized in association with other procedures, and it does not preclude the use of eventual more aggressive future treatments in case of failure.

Since 1984, we have investigated PDT for the treatment of early squamous cell carcinoma arising in the upper aerodigestive tract, the esophagus, and the tracheobronchial tree. Three different photosensitizers were used: hematoporphyrin derivative (HPD), Photofrin II, and tetra(m-hydroxyphenyl)-chlorin (mTHPC). The mTHPC, a second generation photosensitizer, was introduced in our study in 1992, because in preclinical studies it appeared to be superior to HPD and Photofrin II in terms of chemical purity, phototoxicity, and duration of skin photosensitization [17,18]. This paper reports on our overall PDT clinical results for early cancers and summarizes our personal view of the indications and limitations of this treatment.

### MATERIALS AND METHODS

### Patients

From October 1984 to October 1998, 74 patients with biopsy-proven early squamous cell carcinomas (SCC) of the upper aerodigestive tract (UAT), the esophagus, and the bronchi were treated by PDT alone. Ten patients were excluded from the study because the response to treatment could not be evaluated. The main reasons were patient refusal, esophagectomy for other invasive tumors and, in one case, sudden cardiac death 2.5 months after PDT. Among the 64 remaining patients (59 males and 5 females; mean age 58 years; age range of 43–75 years), 39 of them had a single early cancer, whereas 25 had two or more malignancies. The decision to perform PDT was taken by a multidisciplinary team of head and neck surgeons, chemotherapists, and radiotherapists. Photodynamic therapy was mainly chosen as an alternative to more aggressive treatments such as esophagectomy or pulmonary resection. However, in a few cases, patients refused surgical treatment or were clearly not surgical candidates due to severe cardiopulmonary disease. All patients were volunteers and gave written informed consent prior to therapy. Patients with liver dysfunction, as evaluated by liver function tests prior to therapy, were excluded from the study. For each drug, the protocols were approved by the Ethics Committee of the CHUV Hospital in Lausanne.

### Tumors

One hundred and one early squamous cell carcinomas of the oral cavity, pharynx, esophagus, and tracheobronchial tree were treated once or more by PDT. These tumors were detected as second primary malignancies in patients with a primary head and neck cancer. We considered as early carcinomas those that were either *in situ* (Tis), i.e., intraepithelial with no invasion of the basement membrane, or micro-invasive, i.e., with a depth of infiltration less than 2 mm in the oral cavity and the pharynx, with no invasion beyond the muscularis mucosae (T1a) in the esophagus, and no invasion beyond the cartilage and the tunica muscularis in the bronchi. Early tumors can therefore be considered as local lesions with almost no risk of developing lymp node metastasis and a high probability of being completely cured [19,20]. Diagnosis and staging of the tumors were based in all cases on endoscopic morphological criteria as described by Monnier *et al.* [21], as well as on histological examination of biopsy specimens. Since 1993, most of the patients with esophageal cancer underwent endoscopic ultrasonography with 7.5 and 12 MHz probes and more recently with a high resolution 20 MHz miniprobe. Esophageal carcinomas with complete radial extension were not included in the study in order to avoid potential post-treatment stenosis. All of the bronchial tumors were roentgenographically occult as evaluated by chest X-ray and in most of them by computerized tomography.

### Procedure

One hundred and twenty-one PDT treatments were performed because 16 tumors were treated two or three times. First generation photosensitizers (HPD and Photofrin II) were used in 59 cases and mTHPC in 62, respectively.

The HPD was provided freeze-dried by Hoffmann-La Roche and Ciba-Geigy (Basel, Switzerland) and Photofrin II was kindly supplied by Quadra Logic Technology Inc. (Vancouver, BC, Canada) as a lyophilized powder. Both compounds were stored in the dark at 4°C. Shortly before use, they were dissolved in a sterile 5% glucose solution and injected intravenously over a period of 10 min at doses of 3 mg/kg for HPD and 1 or 2 mg/kg for Photofrin II. Irradiation was performed under general anaesthesia 48 or 72 h after the administration of the photosensitizer. Fifty-two of 59 PDTs with HPD and Photofrin II were performed at 630 nm with an argon ion pumped dye laser (Spectra-Physics). The remaining seven PDTs were completed at 514 nm with the same argon ion laser operated in the single-line mode. In the UAT and the bronchi, all of the tumors were treated by surface radiation using microlens and/or cylindrical light distributors [22]. At the beginning of our study, we used circumferential cylindrical light distributors in the esophagus. Over the past eight years, due to post-therapeutic esophageal stenosis, we have only used 180° or 240° windowed cylindrical light distributors with a length of 4 or 6 cm [23,24]. The total light doses ranged from 100 to 180 J/cm$^2$ at both wavelengths. Light was delivered at an intensity varying from 80 to 150 mW/cm$^2$ with an exposure time of about 20 min.

The mTHPC, provided by Scotia Pharmaceuticals Ltd. (Guildford, UK), was dissolved in a solution of 30% polyethylene glycol 400, 20% ethanol, and 50% water. Less than half an hour after its preparation, mTHPC was injected intravenously over a period of 5–10 min through a bacterial filter at doses of 0.3 mg/kg of body weight (twice), 0.15 mg/kg (58 times) or 0.075 mg/kg (twice). Photodynamic therapy was performed under general anaesthesia 20 h (8 cases) and 96 h (54 cases) after the injection of the dye. All but three bronchial and UAT, but only three esophageal irradiations were performed at 652 nm. The remaining cases were treated with green light at 514 nm. Lasers and light distributors were exactly the same as those described above for PDT with HPD and Photofrin II. Most of the 514 nm illuminations were carried out with total light doses of 75–120 J/cm$^2$ and an intensity of 100 mW/cm$^2$; whereas, the majority of the 652 nm irradiations were performed with light doses of 8–12 J/cm$^2$ and an intensity of 150 mW/cm$^2$.

In all cases, the size of the illumination area was larger than the extent of the tumour. The reason for the broad range of light doses applied is that in some cases, the light dose was adapted according to the photosensitizer fluorescence signal measured onto the tumour just before irradiation [25].

### Follow-up

Patients were cautioned to avoid direct sunlight exposure for 4–6 weeks after HPD or Photofrin II administration and for 2 weeks after mTHPC injection. Careful progressive exposure afterwards was recommended during the next 2 months for HPD and Photofrin II and the next 2–4 weeks for mTHPC, respectively. A first endoscopic follow-up was performed 7 or 10 days after PDT to assess the extent of the tumour necrosis macroscopically. Subsequent panendoscopies, done on an outpatient basis with UAT and esophageal staining (Toluidine blue), as well as biopsies and abrasive or wash cytologies, were performed 3 months after the treatment and subsequently twice a year thereafter. Complete response to PDT was defined by the absence of macroscopic signs of tumour at the endoscopic examination and by negative biopsies, cytological washings or brushings. Less than complete response was characterized by the presence of residual tumour on endoscopy or evidence of malignancy on biopsies. Recurrence was considered when a macroscopic or microscopic relapse of the tumour occurred after an apparently complete response at the third month endoscopic control.

### RESULTS

One hundred and one SCCs of the UAT, the esophagus, and the tracheobronchial tree were treated by PDT with HPD, Photofrin II, and mTHPC. Sixteen tumors were located in the oral cavity and the pharynx, 49 in the esophagus, and 36 in the tracheobronchial tree. Maximal tumour extension estimated at endoscopy ranged from 1 to 3 cm in the UAT and from 0.3 to 4 cm in the bronchi. In the esophagus, the superficial longitudinal spread of the tumour varied from 1 to 6 cm with radial extensions between 60° and 200°.

Endoscopic controls 7 or 10 days after PDT revealed not only necrosis of the tumour, but also injuries in the surrounding normal irradiated mucosa. True selectivity was never observed with either photosensitizer administered and the extent of the superficial tissue damage simply matched the geometry of the illuminated area. All of the three dyes showed important inter-individual variations in terms of tumour damage, even in patients treated with the same drug and light parameters. Although difficult to quantify, variations seemed to be more frequently noticed with mTHPC than with HPD or Photofrin II.

Eighty-six (85%) early cancers had a complete response (CR) with follow-up periods of 3 to 99 months (mean 27 months; median 25 months). Nine of these 86 tumors recurred within an interval that ranged from 3 to 34 months. The remaining 15 tumors showed less than CR, with all of them presenting at least partial macroscopic reduction in tumour size. Thus, PDT effectively eradicated 77 (76%) of the cancers evaluated. Examples of early esophageal and bronchial SCC before and after PDT are presented in Figs. 1 and 2, respectively. There was no important difference in CR rates between the three treated sites. Twelve out of 16 (75%) tumors were cured in the UAT, 37 out of 49 (75.5%) in the esophagus, and 28 out of 36 (78%) in the bronchi. The results showed similar PDT efficacy whatever photosensitizer was used. Following treatment with HPD and Photofrin II, 39 of 50 (75%) early cancers achieved a complete response, whereas with mTHPC 38 of 51 (78%) tumors were eradicated. However, as one could expect, the tumour stage is of paramount importance in the efficacy of the treatment even among early cancers. In our study, *in situ* carcinomas had an 89% CR rate, but the ratio fell to 66% in cases of micro-invasive cancers. This lower rate of success is related at least partially to understaging, as documented in esophagectomy specimens, which showed tumoral infiltration of the submucosa in some cases. The results obtained by PDT with the different photosensitizers as a function of the tumour staging and treated site are presented in Tables I and II.

There was no procedure-related mortality. In all cases, intravenous administration of the photosensitizer was well tolerated, despite a slight transient burning sensation localized around the area of injection. There were no episodes of airway compromise after PDT in the bronchi. Following esophageal treatment, some patients reported mild pain on swallowing that was well controlled by short regimens of oral analgesics. The complications of the treatment are listed in Table III. Esophago-tracheal fistulas, which required surgery, occurred in three patients within 3 weeks after red light illumination of lesions located on the anterior wall

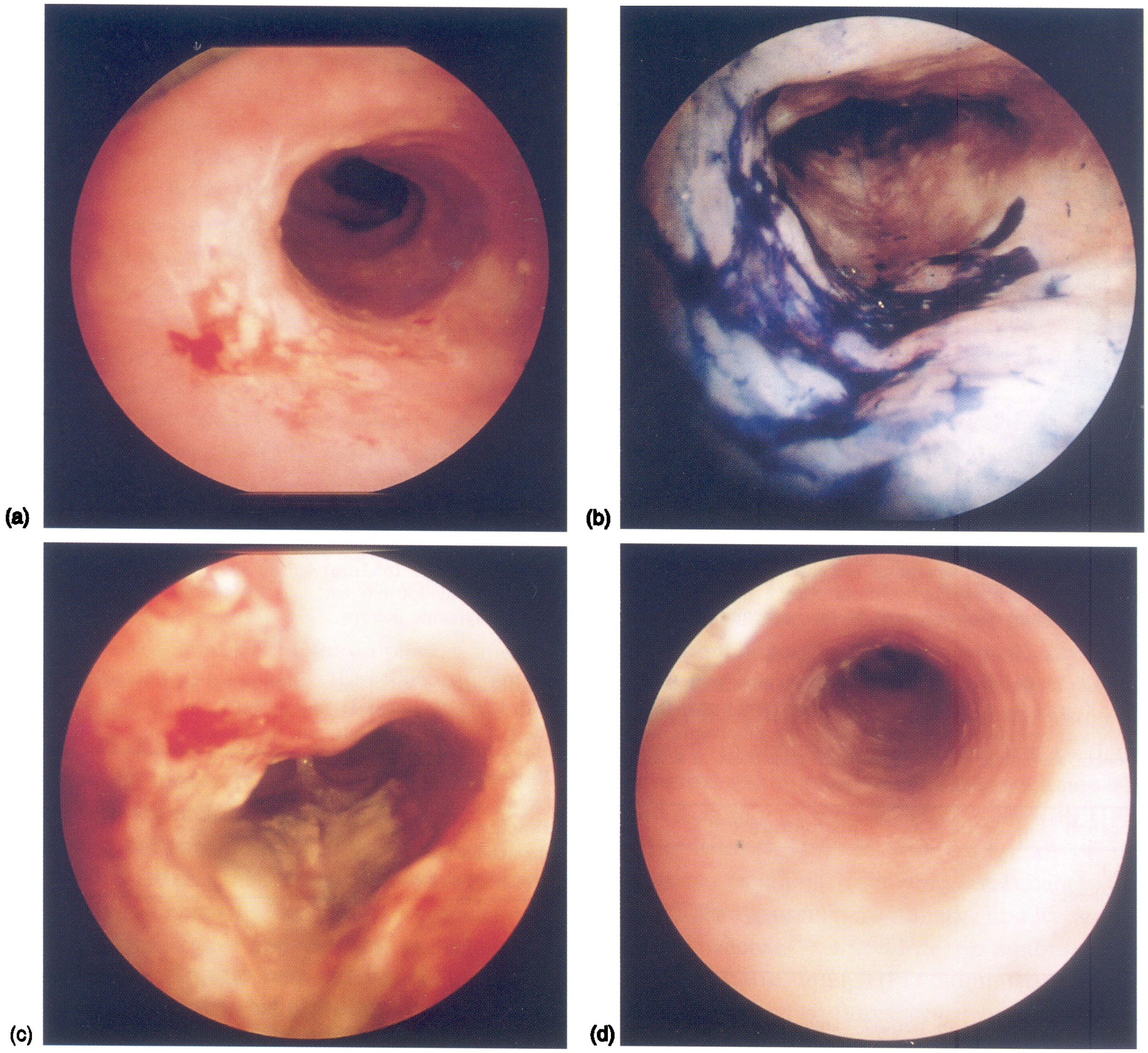

FIGURE 1 Micro-invasive (T1a) squamous cell carcinoma of the esophagus irradiated with green light (514 nm) 4 days after injection of 0.15 mg/kg of tetra(m-hydroxyphenyl) chlorin with a light dose of 160 J/cm$^2$ and an intensity of 100 mW/cm$^2$. (a) Endoscopic view of the tumour before treatment. (b) The same untreated lesion after toluidine blue staining; the extent of the tumour is clearly outlined. (c) Seven days after treatment using a 240° windowed cylindrical light distributor; the radial necrosis corresponds exactly to the irradiated area. (d) Complete response 3 months after photodynamic therapy.

of the mid-esophagus. Two other patients treated with red light for an early tumour in the lower esophagus developed a high-grade fever and pleural effusion, respectively. Both complications were considered as likely transmural necrosis with occult perforation of the esophagus and resolved under antibiotic treatment. Two esophageal stenoses were induced after circumferential (360°) irradiation and were successfully managed by repeated bougienage with a Savary–Gilliard dilator. Twelve patients treated with mTHPC and one with HPD developed skin photosensitization after disregarding the prescribed precautions against sun exposure. All of the mTHPC skin photosensitivity reactions were

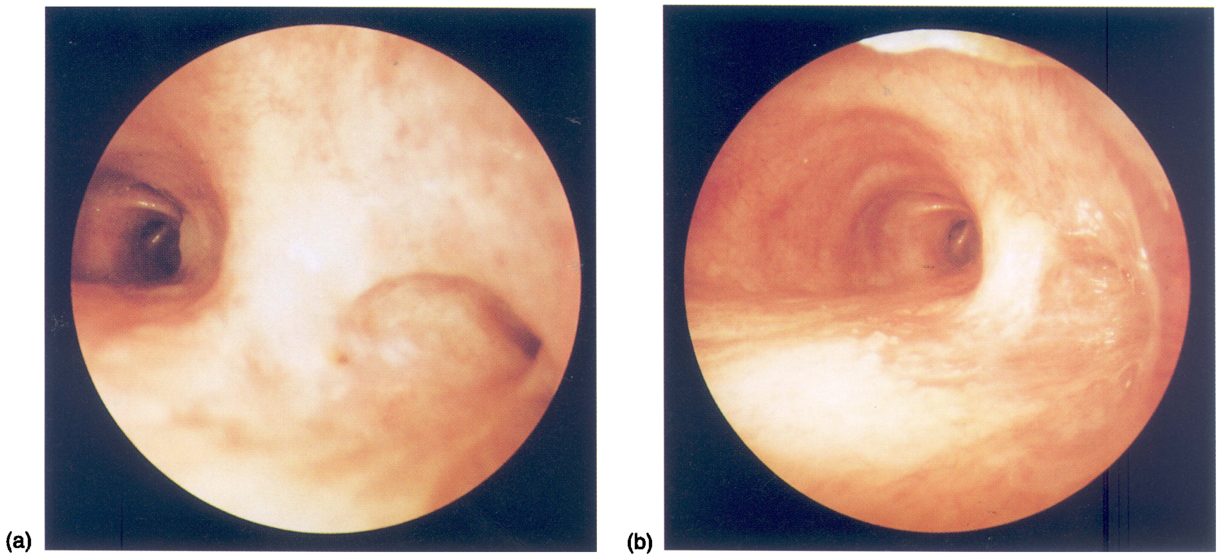

FIGURE 2 *In situ* squamous cell carcinoma of the bronchi treated with red light (652 nm) 4 days after injection of 0.15 mg/kg of tetra(m-hydroxyphenyl) chlorin with a light dose of 12 J/cm$^2$ delivered at 150 mW/cm$^2$. (a) Pre-therapy endoscopic view. (b) No residual tumour is seen on the bronchial spur 3 months after photodynamic therapy.

TABLE I Results of photodynamic therapy with HPD and Photofrin II for 51 early squamous cell carcinomas (SCC) in the upper aerodigestive tract (UAT), the esophagus, and the bronchi: Complete responses with no recurrence as a function of the tumour staging and site

| Location | *In situ* SCC | Micro-invasive SCC | Total |
|---|---|---|---|
| UAT | 3/3 | 4/7 | 7/10 |
| Esophagus | 7/8 | 9/13 | 16/21 |
| Bronchi | 7/8 | 8/12 | 15/20 |
| Total | 17/19 | 21/32 | 38/51 |

TABLE II Results of photodynamic therapy with mTHPC for 50 early squamous cell carcinomas (SCC) in the upper aerodigestive tract (UAT), the esophagus, and the bronchi: Complete responses with no recurrence as a function of the tumour staging and site

| Location | *In situ* SCC | Micro-invasive SCC | Total |
|---|---|---|---|
| UAT | 3/4 | 2/2 | 5/6 |
| Esophagus | 9/10 | 12/18 | 21/28 |
| Bronchi | 11/12 | 2/4 | 13/16 |
| Total | 23/26 | 16/24 | 39/50 |

observed within the first week after injection in patients who did not follow the recommendations for sun protection. The only skin reaction following HPD administration occurred two months after treatment. In all but one case, the skin reaction was moderate and included redness, oedema, and occasionally cutaneous blistering of the hands and face. One patient experienced second degree burns of the face, hand, and forearms after several hours of exposure to sunlight two days after the photosensitizer administration. The patient consequently developed scars after healing.

TABLE III Complications following photodynamic therapy with HPD, Photofrin II, and mTHPC for 101 early cancers of the UAT, the esophagus, and the bronchi

| | |
|---|---|
| Esophagotracheal fistula | 3 |
| Occult esophageal perforation | 2 |
| Esophageal stenosis | 2 |
| Bronchial stenosis | 3 |
| Skin photosensitization | 13 |

### DISCUSSION

Photodynamic therapy is an emerging technique for the treatment of various cancers, including those of the head and neck, the esophagus, and the bronchi. Its benefits are well known and undeniable.

Photodynamic therapy is minimally invasive, non-mutilating and causes few side effects. All of these factors contribute to minimize mortality and morbidity in patients who are already in poor general condition. Since no resistance and no cumulative toxicity have been yet observed, PDT can be repeated multiple times. Photodynamic therapy has also the potential to be used as a neo-adjuvant treatment, as it involves a different curative process. Another somewhat controversial advantage of PDT is the relative selectivity of the photosensitizer for the pathological cells. This property, which allows a selective necrosis of the tumour with preservation of normal tissue, renders PDT more attractive than other thermal techniques, such as laser or electrocautery. This benefit is of paramount importance in head and neck oncology, where standard treatments generate a massive loss of tissue with eventual functional sequelae.

In the head and neck field, the esophagus, and the bronchi, PDT has been applied in both superficial and invasive malignancies [26–31]. However, the use of PDT to palliate advanced lesions has shown only moderate advantages compared to other methods such as thermal YAG laser vaporization or endoprothesis [32,33]. Although PDT temporarily reduces the volume of invasive tumors, its benefit is limited by the severe complications, such as haemorrhage and perforation, related to the procedure [30,34]. Thus, the main interest in using PDT is the cure of superficial cancers. This technique has been successfully employed for early malignancies in the oral cavity, the pharynx, the gastrointestinal tract and the tracheobronchial tree [35–41].

The results of our study confirm that PDT is an efficient treatment for *in situ* and micro-invasive squamous cell carcinomas of the UAT, the esophagus, and the tracheobronchial tree. We have treated more than 100 early tumors and have achieved a complete response in 76% of them, with a mean disease-free follow-up of 27 months. In most cases, a single treatment was sufficient to eradicate the lesion. The relatively low number of early cancers treated in the oral cavity and the pharynx can be explained by the fact that in these particular locations, PDT does not seem to confer relevant advantages in comparison to surgical excision with a $CO_2$ laser [42]. The CR rate after PDT is similar in all three treated sites: UAT, esophagus, and bronchi. This result tends to confirm that efficacy of PDT depends more on an accurate staging of the tumour than on the location of the malignant tissue. The importance of tumour staging in achieving a CR is emphasized by the significantly lower rates obtained in micro-invasive tumors (66%) compared to *in situ* carcinomas (89%).

In all three sites, PDT with mTHPC shows results similar to those obtained with first generation photosensitizers (HPD and Photofrin II). However, mTHPC displays several clinical advantages. Its phototoxicity is much higher as compared to HPD and Photofrin II [18], which implies that lower light doses and shorter illumination times can be used without impairing the treatment efficacy. Brief irradiation times as low as, for example one minute in the bronchi with red light, avoid variations in dosimetry by limiting the movements of light distributors. Skin photosensitivity, as previously reported, is shorter with mTHPC than with HPD or Photofrin II [43]. In our study, all of the cutaneous reactions following treatment with mTHPC always occurred within the first week after injection; whereas, a second degree sunburn happened 2 months after HPD administration. In addition, the higher fluorescence quantum yield displayed by mTHPC enables the important inter-individual variations of tumour fluorescence to be measured just before illumination and thus the light dose delivered to the lesion can be adjusted [25].

Several factors could explain the 24% rate of treatment failures. Undoubtedly, tumour under-staging is one of them. In five cases, esophagectomy was performed, as PDT failed to cure the tumors. Histopathological analysis of the surgical specimens showed submucosal carcinomas (T1b stage) in four cases and limited tumoral invasion of the muscularis propria in the fifth. These cases stress the difficult problem of an accurate differentiation between intramucosal (T1a) and submucosal (T1b)

carcinomas. Since staging of superficial tumors based on morphological criteria is highly dependent on the endoscopist's experience, more complex imaging systems should be developed in the near future. Another possible reason for the less than complete response noticed in some treatments is that tumors are likely to be only partially included in the illumination field. The complex geometry of some lesions, such as bronchial tumors expanding from a spur into different adjacent segmental divisions, probably plays a role in the non-sterilization of the tumors. Esophageal folds likely caused the irregular tumour necrosis that was noticed at the first follow-up endoscopic examination in some cases. In one patient, the frequent sliding of the esophagus due to a hiatal hernia presumably prevented homogenous light delivery. Finally, interpatient variability in the dye concentration at the time of PDT might be responsible for the incomplete eradication of some lesions. Following administration of identical drug and light parameters to different patients, we noticed a wide range of light-induced fluorescence signals in the tissues measured just before PDT, which correlated well with the consequent insufficient or massive degree of tumour necrosis. Therefore, adjusting the light dose to the dye fluorescence signal prior to irradiation could help to minimize the risk of undertreating the lesions.

In our study, all but one major complication occurred following red light illumination. Esophageal perforation with esophagotracheal or esophagomediastinal fistula formation after PDT for invasive cancers has been previously reported [30,44]. These complications are most often related to the extensive necrosis generated by the treatment. However, in the case of early carcinomas, transmural necrosis after PDT is the result of the lack of true selectivity of the photosensitizer's distribution between the tumour and the underlying healthy tissue [11,45–47]. Thus, as the red light penetrates deeply into the tissues, illumination at 630 or 652 nm in the esophagus may not only cause destruction of the early cancer but also of the smooth muscle layers underneath with subsequent perforation of the esophageal wall. Irradiation with green light, which acts more superficially [48], tends to compensate the poor selectivity of the dyes. Since 1992, when we started illuminating early esophageal cancers with 514 nm light, we have not experienced these complications. Furthermore, the use of green light did not adversely affect the efficacy of PDT [49]. In the superficial bronchial cancers as defined here, where by definition the cartilage is not invaded, a transmural necrosis with perforation and fistula formation following PDT is very unlikely to occur. As the bronchial cartilage accumulates only minimal amounts of photosensitizers [50], red light illumination can be safely used. The insufficient therapeutic selectivity of the dyes is underlined by the occurrence in our study of two esophageal stenoses. Even though the tumour to be treated was not circular, a 360° illumination induced a circumferential necrosis that caused a subsequent stricture. Therefore, the application of 180° or 240° windowed light distributors in our study has limited the extent of tissue damage and has prevented the development of further esophageal stenoses.

Our study confirms that PDT is an effective modality for the treatment of early cancer in the UAT, the bronchi, and the esophagus. In addition, the use of optimal light dosimetry with adequate light diffusers renders PDT safer. However, development of new photosensitizers with increased selectivity and limited skin photosensitization, as well as improvement of the light delivery systems should contribute to the expansion and worldwide acceptance of this treatment.

### *Acknowledgements*

This work was supported by the CHUV-EPFL-UNIL fund for collaboration in the area of biomedical technology, the "Fonds de Service" and "Fonds de Perfectionnement" of the Otolaryngology, Head and Neck Surgery Department at the CHUV Hospital, the Swiss National Priority Program in Optics, and the Swiss Commission for Technology and Innovation. We are also grateful to the pharmaceutical companies Hoffmann-La Roche and Ciba-Geigy (Basel, Switzerland), Quadra Logic Technology Inc. (Vancouver, BC, Canada), and Scotia Pharmaceuticals Ltd. (Guilford, UK) for kindly providing the HPD, Photofrin II, and mTHPC, respectively.

### *References*

[1] Schwartz, L.H., Ozsahin, M., Zhang, G.N. *et al.* Synchronous and metachronous head and neck carcinomas. *Cancer* 1994; **74**: 1933–1938.

[2] Dhooge, I.J., De Vos, M. and Van Cauwenberge, P.B. Multiple primary malignant tumors in patients with head and neck cancer: results of a prospective study and future perspectives. *Laryngoscope* 1998; **108**: 250–256.

[3] Gluckman, J.L. and Crissman, J.D. Survival rates in 548 patients with multiple neoplasms of the upper aerodigestive tract. *Laryngoscope* 1983; **93**: 71–74.

[4] Savary, M., Monnier, P., Pasche, R. *et al.* Multiple primary malignancies. *Adv. Otorhinolaryngol.* 1991; **46**: 165–175.

[5] Shons, A.R. and McQuarrie, D.G. Multiple primary epidermoid carcinomas of the upper aerodigestive tract. *Arch. Surg.* 1985; **120**: 1007–1009.

[6] Slaughter, D.P., Southwick, H.W. and Smejkal, W. Field cancerization in oral stratified squamous epithelium. Clinical implications of multicentric origin. *Cancer* 1953; **6**: 963–968.

[7] Levine, B. and Nielsen, E.W. The justification and controversies of panendoscopy – a review. *Ear Nose Throat J.* 1992; **71**: 335–340.

[8] Edell, E.S. and Cortese, D.A. Photodynamic therapy in the management of early superficial squamous cell carcinoma as an alternative to surgical resection. *Chest* 1992; **102**: 1319–1322.

[9] Sibille, A., Lambert, R., Souquet, J.C. *et al.* Long-term survival after photodynamic therapy for esophageal cancer. *Gastroenterology* 1995; **108**: 337–344.

[10] Kato, H., Horai, T., Furuse, K. *et al.* Photodynamic therapy for cancers: a clinical trial of porfimer sodium in Japan. *Jpn. J. Cancer Res.* 1993; **84**: 1209–1214.

[11] Monnier, Ph., Savary, M., Fontolliet, Ch. *et al.* Photodetection and photodynamic therapy of early squamous cell carcinomas of the pharynx, esophagus and tracheo-bronchial tree. *Lasers Med. Sci.* 1990; **5**: 149–169.

[12] Monnier, Ph., Fontolliet, Ch., Wagnieres, G. *et al.* Further appraisal of PDI and PDT of early squamous cell carcinomas of the pharynx, esophagus and bronchi. In: Spinelli, P., Dal Fante, M. and Marchesini, R. (Eds.) *Photodynamic Therapy and Biomedical Lasers.* Excerpta Medica. International Congress Series 1992; **1011**: 7–14.

[13] Grant, W.E., Hopper, C., Speight, P.M. *et al.* Photodynamic therapy of malignant and premalignant lesions in patients with "field cancerization" of the oral cavity. *J. Laryngol. Otol.* 1993; **107**: 1140–1145.

[14] Biel, M.A. Photodynamic therapy and the treatment of head and neck neoplasia. *Laryngoscope* 1998; **108**: 1259–1268.

[15] Dougherty, T.J., Gomer, C.J., Henderson, B.W. *et al.* Photodynamic therapy. *J. Natl. Cancer Inst.* 1998; **90**: 889–905.

[16] Fisher, A.M.R., Murphree, A.L. and Gomer, C.J. Clinical and preclinical photodynamic therapy. *Lasers Surg. Med.* 1995; **17**: 2–31.

[17] Berenbaum, M.C., Akande, S.L., Bonnett, R. *et al.* Meso-tetra(hydroxyphenyl) porphyrins, a new class of potent tumour photosensitizers with favourable selectivity. *Br. J. Cancer* 1986; **54**: 717–725.

[18] Berenbaum, M.C., Bonnett, R., Chevretton, E.B. *et al.* Selectivity of meso-tetra(hydroxyphenyl)porphyrins and chlorins and Photofrin in causing photodamage in tumour, skin, muscle and bladder. The concept of cost-benefit in analysing the results. *Laser Med. Sci.* 1993; **8**: 235–243.

[19] Bogomoletz, W.V., Molas, G., Gayet, B. *et al.* Superficial squamous cell carcinoma of the esophagus. A report of 76 cases and review of the literature. *Am. J. Surg. Pathol.* 1989; **13**: 535–546.

[20] Woolner, L.B., Fontana, R.S., Cortese, D.A. *et al.* Roentgenographically occult lung cancer: pathologic findings and frequency of multicentricity during a 10-year period. *Mayo Clin. Proc.* 1984; **59**: 453–466.

[21] Monnier, Ph., Fontolliet, Ch. and Ollyo, J.B. Endoscopic findings of 100 early-stage esophageal cancers. *Diagn. Therap. Endosc.* 1994; **1**: 83–92.

[22] Wagnieres, G., Monnier, Ph., Savary, M. *et al.* Photodynamic therapy of early cancer in the upper aerodigestive tract and bronchi: instrumentation and clinical results. *Proc. Soc. Photo-Opt. Instr. Eng.* 1990; **IS6**: 249–271.

[23] Van den Bergh, H., Mizeret, J., Theumann, J.F. *et al.* Light distributors for photodynamic therapy. *SPIE Proceedings Series*, Bellingham WA, USA 1996; **2631**: 173–198.

[24] Van den Bergh, H. On the evolution of some endoscopic light delivery systems for photodynamic therapy. *Endoscopy* 1998; **30**: 392–407.

[25] Braichotte, D.R., Savary, J.F., Monnier, Ph. *et al.* Optimizing light dosimetry in photodynamic therapy of early stage carcinomas of the esophagus using fluorescence spectroscopy. *Lasers Surg. Med.* 1996; **19**: 340–346.

[26] Biel, M. Photodynamic therapy and the treating of head and neck cancers. *J. Clin. Laser Radiat. Surg.* 1996; **14**: 239–244.

[27] Schweitzer, V.G. Photodynamic therapy for treatment of head and neck cancer. *Otolaryngol. Head Neck Surg.* 1990; **102**: 225–232.

[28] McCaughan, J.S. Jr., Hawley, P.C., Bethel, B.H. *et al.* Photodynamic therapy of endobronchial malignancies. *Cancer* 1988; **62**: 691–701.

[29] Balchum, O.J., Doiron, D.R. and Huth, G.C. Photoradiation therapy of endobronchial lung cancers employing the photodynamic action of hematoporphyrin derivative. *Lasers Surg. Med.* 1984; **4**: 13–30.

[30] McCaughan, J.S. Jr., Nims, T.A., Guy, J.T. *et al.* Photodynamic therapy for esophageal tumors. *Arch. Surg.* 1989; **124**: 74–80.

[31] Marcon, N.E. Photodynamic therapy and cancer of the esophagus. *Semin. Oncol.* 1994; **21**: S20–S23.

[32] Lam, S. Photodynamic therapy in lung cancer. *Semin. Oncol.* 1994; **21**: S15–S19.

[33] Bown, S.G. Photodynamic therapy in gastroenterology. Current status and future prospects. *Endoscopy* 1993; **25**: S683–S686.

[34] Cortese, D.A. and Kinsey, J.H. Endoscopic management of lung cancer with hematoporphyrin derivative phototherapy. *Mayo Clinic Proc.* 1982; **57**: 543–547.

[35] Feyh, J., Goetz, A., Muller, W. *et al.* Photodynamic therapy in head and neck surgery. *J. Photochem. Photobiol. B. Biol.* 1990; **7**: 353–358.

[36] Wenig, B.L., Kurtzman, D.M., Grossweiner, L. *et al.* Photodynamic therapy in the treatment of squamous cell carcinoma of the head and neck. *Arch. Otolaryngol. Head Neck Surg.* 1990; **116**: 1267–1270.

[37] Furuse, K., Fukuoka, M., Kato, H. *et al.* Prospective phase II study on photodynamic therapy with photofrin II for centrally located early-stage lung cancer. The Japan Lung Center. *J. Clin. Oncol.* 1993; **11**: 1852–1857.

[38] Kato, H., Okunaka, T. and Shimatani, H. Photodynamic therapy for early stage bronchogenic carcinoma. *J. Clin. Laser Med. Surg.* 1996; **14**: 235–238.

[39] Grosjean, P., Savary, J.F., Wagnieres, G. *et al.* Tetra-(m-hydroxyphenyl) chlorin clinical photodynamic therapy of early bronchial and oesophageal cancers. *Lasers Med. Sci.* 1996; **11**: 227–235.

[40] Grosjean, P., Savary, J.F., Mizeret, J. *et al.* Photodynamic therapy for cancer of the upper aerodigestive tract using tetra(m-hydroxyphenyl) chlorin. *J. Clin. Laser Med. Surg.* 1996; **14**: 281–287.

[41] Savary, J.F., Grosjean, P., Monnier, Ph. *et al.* Photodynamic therapy of early squamous cell carcinomas of the esophagus: a review of 31 cases. *Endoscopy* 1998; **30**: 258–265.

[42] Davis, R.K. Photodynamic therapy in head and neck cancer. *Adv. Otorhinolaryngol.* 1995; **49**: 58–62.

[43] Savary, J.F., Monnier, Ph., Wagnieres, G. *et al.* Preliminary clinical studies of photodynamic therapy with meso-tetra-hydroxyphenyl chlorin (m-THPC) as a photosensitizing agent for the treatment of early pharyngeal, esophageal and bronchial carcinomas. *SPIE Proceedings Series.* Bellingham WA, USA 1993; **2078**: 330–340.

[44] Thomas, R.J., Abbot, M., Bhathal, P.S. *et al.* High-dose photoirradiation of esophageal cancer. *Ann. Surg.* 1987; **206**: 193–199.

[45] Andrejevic, S., Savary, J.F., Monnier, Ph. *et al.* Measurements by fluorescence microscopy of the time dependent mesotetra-hydroxyphenyl chlorin distribution in healthy tissues and chemically induced early squamous cell carcinoma of the Syrian hamster cheek pouch. *J. Photochem. Photobiol. B. Biol.* 1996; **36**: 143–151.

[46] Braichotte, D., Wagnieres, G., Bays, R. *et al.* Clinical pharmacokinetic studies of Photofrin by fluorescence spectroscopy in the oral cavity, the esophagus. and the bronchi. *Cancer* 1995; **75**: 2768–2778.

[47] Braichotte, D., Savary, J.F., Glanzmann, T. *et al.* Clinical pharmacokinetic studies of tetra(meta-hydroxyphenyl) chlorin in squamous cell carcinoma by fluorescence spectroscopy at 2 wavelengths. *Int. J. Cancer* 1995; **63**: 198–204.

[48] Bays, R., Wagnieres, G., Robert, D. *et al.* Clinical determination of tissue optical properties by endoscopic spatially resolved reflectometry. *Appl. Optics* 1996; **35**: 1756–1766.

[49] Grosjean, P., Wagnieres, G., Fontolliet, Ch. *et al.* Clinical photodynamic therapy for superficial cancer in the esophagus and the bronchi: 514 nm compared with 630 nm light irradiation after sensitization with Photofrin II. *Brit. J. Cancer* 1998; **77**: 1989–1995.

[50] Smith, S.G., Bedwell, J., MacRobert, A.J. *et al.* Experimental studies to assess the potential of photodynamic therapy for the treatment of bronchial carcinomas. *Thorax* 1993; **48**: 474–480.

